# Physiological Mechanisms of Improved Smut Resistance in Sugarcane Through Application of Silicon

**DOI:** 10.3389/fpls.2020.568130

**Published:** 2020-11-05

**Authors:** Quanqing Deng, Jia Wu, Jianwen Chen, Wankuan Shen

**Affiliations:** ^1^College of Agriculture, South China Agricultural University, Guangzhou, China; ^2^Scientific Observing and Experimental Station of Crop Cultivation in South China, Ministry of Agriculture, Guangzhou, China

**Keywords:** silicon, sugarcane smut, *Sporisorium scitamineum*, disease resistance, physiological mechanism

## Abstract

Sugarcane smut caused by *Sporisorium scitamineum* is a severe, global sugarcane disease with severe economic losses and is difficult to prevent. To explore more effective control techniques for smut, the effects and physiological mechanism of silicon (Si) on smut resistance in two smut-susceptible cultivars, ROC22 and Badila, were investigated. The results show that Si application significantly enhances smut resistance in ROC22 and Badila, and the incidence of sugarcane smut decreased by 11.57–22.58% (ROC22) and 27.75–46.67% (Badila). The incidence of smut is negatively correlated with the amount of Si applied and the Si content in sugarcane leaves, stems, and roots (highly significantly negatively correlated with stem Si content). Under *S. scitamineum* stress, the activities of pathogenesis-related enzymes, chitinase and β-1,3-glucanase, secondary metabolism-related enzymes such as polyphenoloxidase (PPO) and phenylalanine-ammonia-lyase (PAL), and the contents of secondary metabolites, total soluble phenol, and lignin in sugarcane leaves treated with Si were significantly higher than those without Si (CK). The results also demonstrated that the content of malondialdehyde (MDA) and hydrogen peroxide (H_2_O_2_), the superoxide dismutase (SOD) activity of sugarcane leaves treated with Si increased in the seedling and tillering stages, and the peroxidase (POD) activity decreased in the seedling stage, which caused the accumulation of reactive oxygen species (ROS) that in turn triggered defense responses. Moreover, MDA and H_2_O_2_ levels decreased, and the activities of SOD and POD increased at the jointing stage, which was beneficial to the removal of excessive ROS. Collectively, these results suggest that Si modulates pathogenesis-related protein activity, secondary metabolism, and active oxygen metabolism of sugarcane that positively regulate resistance to smut. This study is the first to reveal the physiological mechanism of Si in improving smut resistance in sugarcane, and the results provide a theoretical basis for the development of Si fertilizers to control sugarcane smut.

## Introduction

Sugarcane (*Saccharum* spp. hybrids) is an important sugar crop and a renewable biomass energy crop with great development potential. China is the third largest sugarcane producer in the world, following Brazil and India. Sugarcane smut caused by *Sporisorium scitamineum* is an important global sugarcane fungal disease. Since the first report of sugarcane smut in Natal, South Africa, in 1877, the disease has now become a global sugarcane disease (Croft et al., 2008; Bhuiyan and Croft, 2015; Su et al., 2016). In the past 20 years, smut has become one of the most economically harmful sugarcane diseases in mainland China. Currently, the main sugarcane cultivars in China are infected with smut, and the incidence in the field ranges from 10% to 20%, severely affecting yield and quality (Que et al., 2012, 2014; Shen et al., 2014a). The mycelium of *S. scitamineum* infects sugarcane buds and spreads through intercellular hyphae to the rest of the cane. In addition, the sugarcane stalk rind is thick and rich in wax, preventing common chemicals to penetrate the sugarcane stem to kill pathogens, thereby preventing the effective control of sugarcane smut (Shen et al., 2012). Disease-resistance breeding is the main way to control crop diseases. However, due to the pathogenicity or physiological race differentiation of *S. scitamineum* and sugarcane being an allopolyploid with complex inheritance and long breeding cycle, it is not easy to succeed in sugarcane breeding for smut resistance (Shen and Deng, 2011; Que et al., 2012; Su et al., 2016). Therefore, it is necessary to establish a novel and effective method to manage sugarcane smut.

Silicon (Si) is a common soil mineral that has been shown to improve disease resistance in crops. Si can promote resistance to rice blast (*Magnaporthe grisea*), wheat powdery mildew (*Blumeria graminis* f. sp. *tritici*), rice brown spot (*Cochliobolus miyabeanus*), rice bacterial blight (*Xanthomonas oryzae* pv. *oryzae*), tomato bacterial wilt (*Ralstonia solanacearum*), and other fungal or bacterial diseases (Rodrigues et al., 2005; Cai et al., 2008; Rémus-Borel et al., 2009; Kiirika et al., 2013; Bockhaven et al., 2015; Liu et al., 2016). In addition, the preliminary mechanism of Si improving disease resistance has also been reported in some crops. For instance, wheat powdery mildews caused by *Blumeria graminis* f. sp. *tritici* was especially well controlled by Si regulating methyl trans-aconitate production (Rémus-Borel et al., 2009). Si can improve rice resistance to rice blast caused by *Magnaporthe grisea* through actively changing the accumulation of glucanase, peroxidase, and pathogenesis-related transcripts (Rodrigues et al., 2005).

Sugarcane is a moderate Si-accumulating crop, with absorption rates higher than that for other minerals except potassium (Meyer and Keeping, 2005). Si plays an important role in the growth and development of sugarcane, thereby improving the quality of sugarcane, and more importantly, enhancing resistance to biological and abiotic stresses (Meyer and Keeping, 2005; Huang et al., 2011; Camargo et al., 2013; Jiang et al., 2014; Bhuiyan and Croft, 2015; Lu et al., 2016; Ramouthar et al., 2016). There are a few reports on the improvement of sugarcane resistance to disease by Si application. Camargo et al. (2013) used Si fertilizer to control brown rust (*Puccinia melanocephala*) in three typical soil types of sugarcane through 1 year of new planting and 2 years of ratooning experiments in Brazil, and the results showed that Si fertilizer reduce the incidence of brown rust in different degrees (the control effect was 20–59%). In the sand sugarcane area of Australia, a blast furnace slag (Si content 14–18%) was used as Si source in the field plot experiment for three consecutive years. The preliminary results showed that blast furnace slag significantly reduces the incidence of sugarcane smut in the field (various sugarcane genotypes have different control effects) and promote the growth of sugarcane, but the mechanism was not investigated (Bhuiyan and Croft, 2015). Therefore, this study elucidated the physiological mechanism of Si application to improve smut resistance in sugarcane for the first time, as well as provide a theoretical basis for the development of novel strategies of Si application to control smut in sugarcane.

## Materials and Methods

### Plant Materials

Two pot experiments were conducted from June to October in 2016 and 2017. Seedcanes of the predominant sugarcane cultivar ROC22 (highly susceptible to smut) and the main chewing cane cultivar Badila (*S. offcinarum* L., highly susceptible to smut) were obtained from the resource garden of the sugarcane breeding base of South China Agricultural University, where sugarcane smut is uncommon. Sugarcane stalks that were carefully inspected to ensure that these were free of smut and other systemic diseases were cut into single buds.

### Teliospore Collection and Inoculation

Sugarcane smut teliospores were collected from smut whips of ROC22 from Zhanjiang, Guangdong, which is the main sugarcane production area in China (Deng et al., 2018). The germination rate of the teliospores before inoculation was tested, and the germination rates were all >90% (Bhuiyan et al., 2012; Shen et al., 2014b). In this study, the dip inoculation method was used. First, the tested qualified teliospores were prepared as a spore suspension at a concentration of 5 × 10^6^ spores ml^–1^ (i.e., 2 g of teliospore powder mixed with 1 L of ddH_2_O). After the healthy sugarcane single buds were soaked in the spore suspension for 30 min, these were then sealed in a plastic bag to retain its moisture, and then germinated at 28°C for 24 h before planting (Shen et al., 2014c).

### Experimental Design

Samples of the 20-cm-deep plow layer soil of sugarcane field (sugarcane breeding base of South China Agricultural University) were collected and air-dried in the greenhouse. After mixing, these were used as pot planting test soil (2016: total nitrogen, 0.88 g kg^–1^; total phosphorus, 0.97 g kg^–1^; total potassium, 19.35 g kg^–1^; alkali hydrolyzed nitrogen, 72.63 mg kg^–1^; effective phosphorus, 48.17 mg kg^–1^; available potassium, 53.43 mg kg^–1^; effective Si, 42.39 mg kg^–1^; 2017: total nitrogen, 0.85 g kg^–1^, total phosphorus, 0.84 g kg^–1^; total potassium, 20.53 g kg^–1^; alkali hydrolyzed nitrogen 79.26 mg kg^–1^; effective phosphorus 64.43 mg kg^–1^; available potassium 57.87 mg kg^–1^; effective Si 55.23 mg kg^–1^). The test soil was Si deficient as the critical soil effective Si content was 105–120 mg/kg (Liang et al., 2015). Plastic pots with respective height, upper diameter, and lower diameter of 32.0, 33.5, and 28.0 cm were used for the test; the bottom of each pot has three holes (1.0 cm in diameter) for draining excess water in the soil, and the soil weight per pot was 10 kg (approximately two thirds of the pot height, and the soil moisture is 10%). Quantitative exogenous silicon (ESi) (Na_2_SiO_3_⋅5H_2_O, 19.3–22.8% available Si, Tianjin Damao Chemical Reagent Factory, China) was added to the soil, mixed with a small amount of water, and then placed in the greenhouse for more than 2 weeks for further testing (Camargo et al., 2013).

Two pot experiments involved single-factor Si applications, i.e., *S. scitamineum* (sugarcane smut teliospores) inoculation. Four inoculated single buds were planted per pot and placed in the greenhouse that was located at the sugarcane breeding base of South China Agricultural University. Three Si treatments, three pots per treatment, with three replications were prepared for the ROC22 genotypes in the 2016 experiment: (1) CK1 (no ESi application); (2) Sis5 (5 g ESi barrel^–1^); and (3) Sis10 (10 g ESi barrel^–1^) (Huang et al., 2011). Four Si treatments, three barrels per treatment, with three replications were prepared for the Badila genotypes in the 2017 experiment: (1) CK2 (no ESi application); (2) Sis15 (15 g ESi barrel^–1^); (3) Sis30 (30 g ESi barrel^–1^); and (4) Sis45 (45 g ESi barrel^–1^) (Huang et al., 2011). The two pot experiments were arranged in a randomized complete block design.

### Disease Investigation and Control Effect Assessment

When sugarcane smut whip was found for the first time, the first disease statistics was performed. The number of plants and smut whips was determined and recorded once a week (including plants with early symptoms) until no new smut whips appeared (about 3 months). At the end of each investigation, the recorded diseased plants were labeled to avoid repeated investigation, and the recorded smut whips were wrapped with a filter bag to prevent the spread of teliospores. After the experiment, the incubation period, incidence rate, and control effect of sugarcane smut were calculated. The incubation period of sugarcane smut is the number of days from inoculation to the first occurrence of obvious symptoms (whip). The incidence of diseased plants was calculated as follows: (diseased plants/total number of plants) × 100% (Deng et al., 2018). The control effect (%) was calculated as follows: (disease incidence of CK – disease incidence of treatment)/disease incidence of CK × 100%.

### Measurement of Total Si Content

After statistical analysis of smut incidence, the roots, stems, and leaves were sampled separately from three plants per barrel and dried at 110°C for 30 min and then at 80°C until a constant mass was reached. The dried samples were ground with a high-speed grinder and passed through a 100-mesh sieve. The sample powder was kept dry for later use. The high-temperature alkaline melting method was used to measure the total Si content (Fox et al., 1969; Dai et al., 2005).

### Measurement of Defense-Related Physiological Parameters

Nine leaves (the first fully expanded functional leaf under the heart leaf) were randomly sampled from each treatment at the seedling, tillering, and jointing stages (three replications, and three leaves per replication) and stored in an ultralow-temperature refrigerator at −80°C until analysis. To determine the activity of chitinase and β-1,3-glucanase in leaves, the amount of reducing sugar was determined using the dinitrosalicylic acid (DNSA) method as described by Miller (1959) and Kestwal et al. (2011). The extraction method of the crude enzyme solution of pathogenesis-related proteins was performed according to Bagal-Kestwal et al. (2009). The reaction substrate of the 1% chitin colloidal suspension was prepared according to Imanaka et al. (2001). Polyphenoloxidase (PPO) activity was investigated according to Meng et al. (2012). Phenylalanine-ammonia-lyase (PAL) activity was assessed as described by Lisker et al. (1983). Total soluble phenol and lignin determination was based on the method of Rodrigues et al. (2005). MDA content was measured according to Zeng et al. (2017). H_2_O_2_ was determined using the iodometric method (Junglee et al., 2014). SOD, POD, and CAT activities were assessed according to Zhang et al. (2017).

### Statistical Analysis

All of the data were expressed as the mean ± SE. Microsoft Excel 2013 (Microsoft Corp., Redmond, WA, United States) was used for data processing, and SPSS version 21 software (IBM Corp., Armonk, NY, United States) was used for one-way ANOVA and correlation analysis. Differences between parameters were evaluated using Duncan’s method, and differences with *P* ≤ 0.05 were considered to be statistically significant. Origin 9.0 software was used for histogram plotting (i.e., Figures 2–5).

## Results

### Effect of Si Application on Sugarcane Smut

**FIGURE 1:**
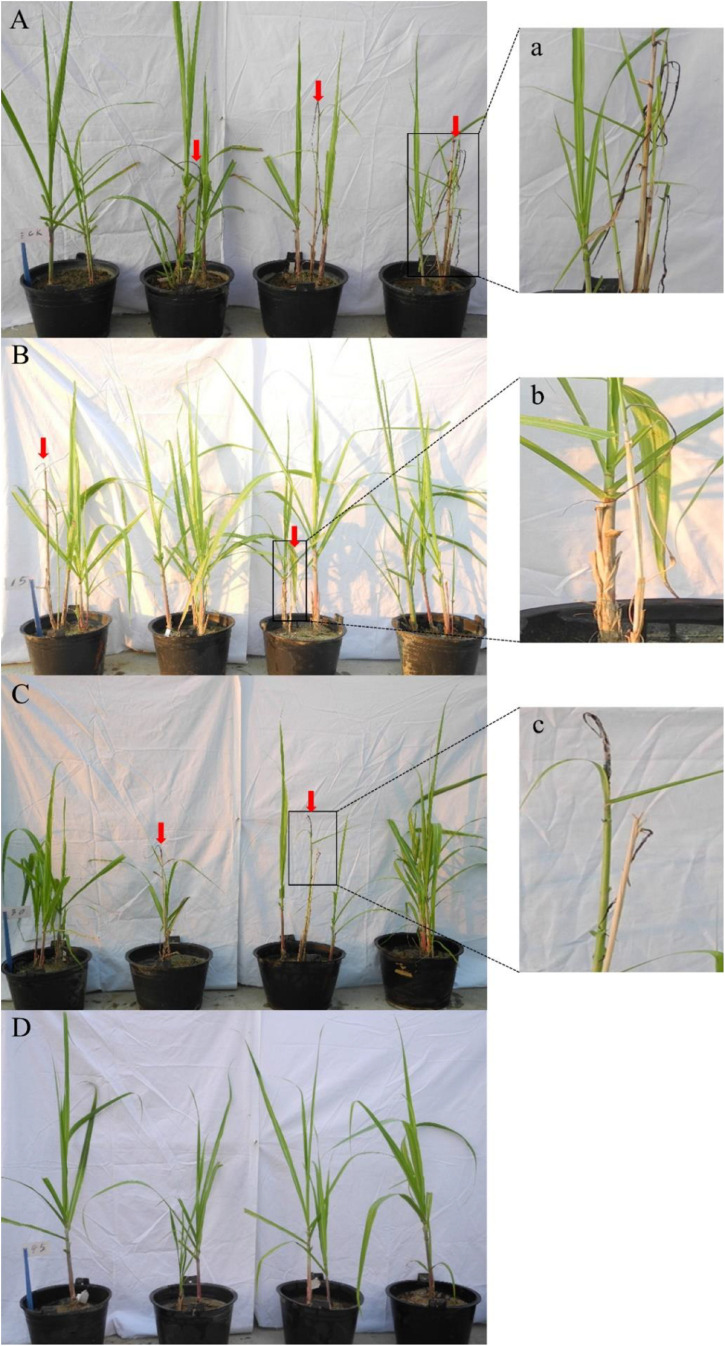
Effect of silicon application on the occurrence of smut in sugarcane cultivar Badila. **(A)** CK2, no exogenous silicon (ESi) application; **(B)** Sis15, 15 g ESi barrel^–1^; **(C)** Sis30, 30 g ESi barrel^–1^; **(D)** Sis45, 45 g ESi barrel^–1^. **(a,b,c)**: enlarged views of local smut whip for **(A–C)**. The red arrow refers to smut whips.

In the 2016 experiment (ROC22), the incubation period of the three treatments was 71–78 days, that of Sis10 treatment was 78 days, and that of the other two treatments was 71 days. In the 2017 experiment (Badila), the incubation period of the four treatments was 60–∞ days, that of CK2 was 60 days, that of Sis30 was longer than that of Sis15, and that of Sis45 did not exhibit smut whip, indicating that the incubation period of Si treatment was longer than that of non-Si treatment, and the incubation period was prolonged with increasing of Si application (Table 1). In the 2016 experiment, the highest incidence of sugarcane smut was CK1 (40.64%), and the lowest was Sis10 (18.06%), with significant difference (*P* ≤ 0.05). Smut incidence of Sis15, Sis30, and Sis45 was significantly (*P* ≤ 0.05) lower than that of CK2; the highest incidence was observed in CK2 (46.67%), the lowest in Sis45 (0), and the incidence of Sis15 and Sis30 were, respectively, 18.92% and 15.0% in the 2017 experiment. In the two-year experiment, the effect of Si application on sugarcane smut was significant (*P* ≤ 0.05) (Figure 1 and Table 1); in the 2016 experiment, the control effect decreased in the following order: Sis10 (56.02%) > Sis5 (29.26%) > CK1 (0), and was determined to be statistically significant (*P* ≤ 0.05). In the 2017 experiment, the control effect decreased in the following order: Sis45 (100.00%) > Sis30 (70.00%) > Sis15 (55.56%) > CK2 (0). In addition, the control effect of Sis45 was significantly (*P* ≤ 0.05) higher than that of Sis15.

**TABLE 1:** Effect of silicon on disease latency, disease incidence, and control effect under *Sporisorium scitamineum* stress.

Experiment	Cultivar	Treament^1^	Disease latency^2^ (days)	Disease incidence (%)	Control effect (%)
2016	ROC22	CK1	71	40.64 ± 2.03a	0a
		Sis5	71	29.07 ± 5.52ab	29.26 ± 10.15b
		Sis10	78	18.06 ± 3.67b	56.02 ± 7.22c
2017	Badila	CK2	60	46.67 ± 3.33a	0a
		Sis15	67	18.92 ± 1.07b	55.56 ± 13.87b
		Sis30	74	15.00 ± 7.64b	70.00 ± 15.27cb
		Sis45	∞	0c	100.00c

*^1^CK1, no ESi application; Sis5, 5 g ESi barrel^–1^; Sis10, 10 g ESi barrel^–1^; CK2, no ESi application; Sis15, 15 g ESi barrel^–1^; Sis30, 30 g ESi barrel^–1^; Sis45, 45 g ESi barrel^–1^. ^2^∞, Sis45 was not showing smut whip, which means that the incubation period is infinite. Data are expressed as mean ± SE. Different letters in the same column and genotype show significant differences at 0.05 level of probability according to Duncan’s multiple comparison test among treatments.*

### Relationship Between Si Content and Resistance to Sugarcane Smut

**FIGURE 2:**
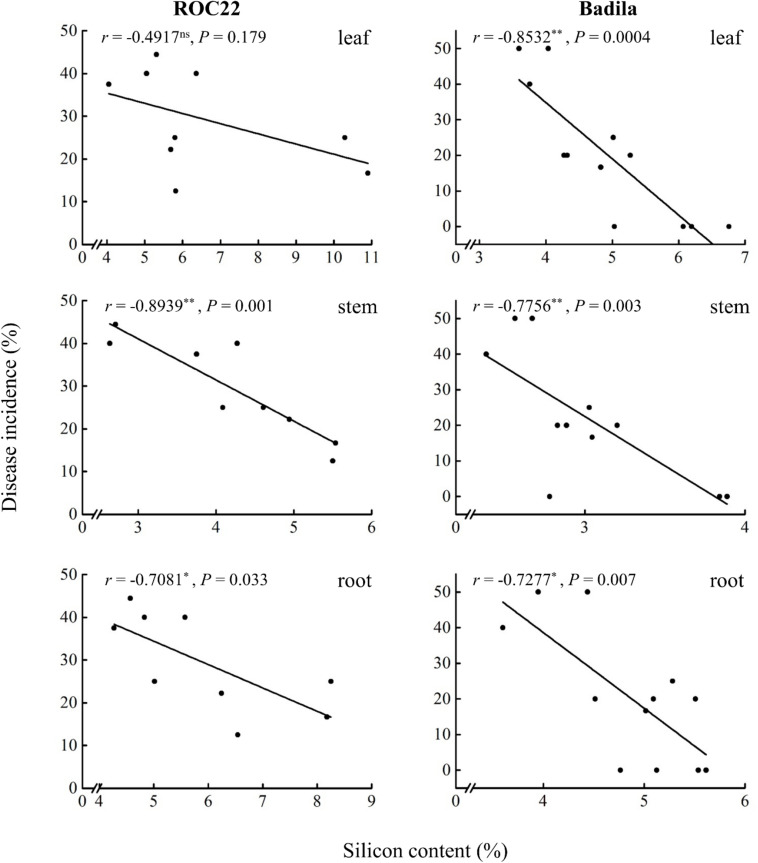
Correlation between silicon content in the leaves, stems, and roots of sugarcane and smut incidence. Levels of correlation: ns, non-significant; *≤ 0.05 and **≤ 0.01. *r*, correlation coefficient. *P*, *P*-value.

The Si content in the leaves, stems, and roots of sugarcane cultivars ROC22 and Badila is negatively correlated with the incidence of smut. The negative correlation between Si content in the stems and the incidence of sugarcane smut is highly significant (*P* ≤ 0.05) in the two varieties (Figure 2). In ROC22, the higher the Si content in the stems and roots, the lower the incidence of smut, and the correlation coefficients were −0.8939^∗∗^ and −0.7081^∗^, respectively, whereas the Si content in the leaves and the incidence of sugarcane smut showed a non-statistically significant (*P* ≤ 0.05) negative correlation (*r* = −0.4971). The correlation between Si content and smut resistance in decreasing order is as follows: stem > root > leaf, which shows that Si application could significantly (*P* ≤ 0.05) reduce smut incidence in ROC22, and the higher the Si content in the stems, the more significant the resistance to smut. In Badila, the higher the Si content in the leaves, stems, and roots, the lower the incidence of sugarcane smut, and the correlation coefficients were −0.8532^∗∗^, −0.7756^∗∗^, and −0.7277^∗^, respectively. The correlation between Si content and smut resistance in decreasing order is as follows: leaf > stem > root, which illustrates that Si application could significantly (*P* ≤ 0.05) reduce smut incidence in Badila, and the higher the Si content in the leaves and stems, the more significant is the resistance to smut.

### Activities of Pathogenesis-Related Enzymes in Sugarcane Leaves

**FIGURE 3:**
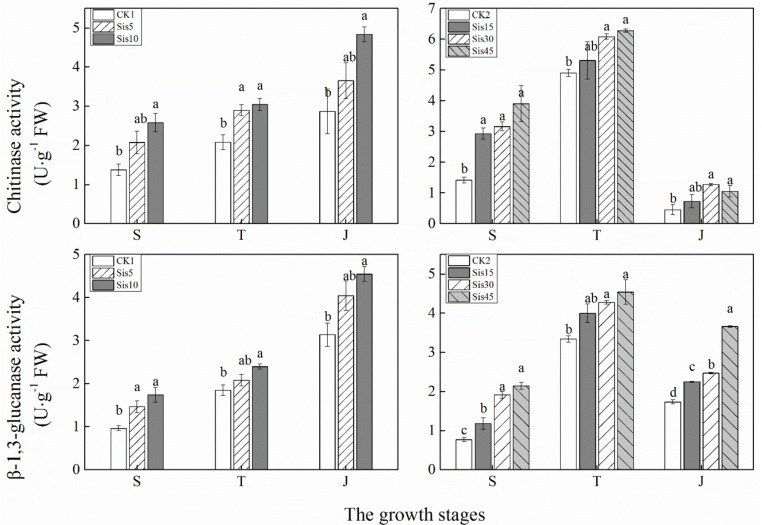
Effects of silicon application on chitinase and β-1,3-glucanase activities in sugarcane leaves under *Sporisorium scitamineum* stress. Various lowercase letters represent significant differences at the *P* level of 0.05. S, seedling stage; T, tillering stage; J, jointing stage.

In ROC22, the activities of chitinase and β-1,3-glucanase in the leaves with different Si applications showed increased with growth (Figure 3). The activity of these two enzymes increased with greater Si applications in three growth stages (seedling, tillering, and jointing). Compared with CK1, chitinase activity in Sis10 in the three growth stages and Sis5 at tillering stage both significantly increased (*P* ≤ 0.05), and β-1,3-glucanase activity in Sis10 in the three growth stages and Sis5 in the seedling stage increased to a significant level (*P* ≤ 0.05).

In Badila, with growth, chitinase and β-1,3-glucanase activities in the sugarcane leaves using different Si application rates initially increased and then decreased, with the highest activities at the tillering stage (Figure 3). The activities of chitinase and β-1,3-glucanase in the three growth stages (seedling, tillering, and jointing) increased with the increase in Si application. Compared with CK2, chitinase activity in Sis15, Sis30, and Sis45 at the seedling stage and Sis30 and Sis45 at the tillering and jointing stages significantly increased (*P* ≤ 0.05). In addition, the activities of β-1,3-glucanase in the treatment of Sis15, Sis30, and Sis45 at the seedling and jointing stages and Sis30 and Sis45 at the tillering stage all significantly increased (*P* ≤ 0.05).

### Secondary Metabolism-Related Enzymes and Metabolites in Sugarcane Leaves

**FIGURE 4:**
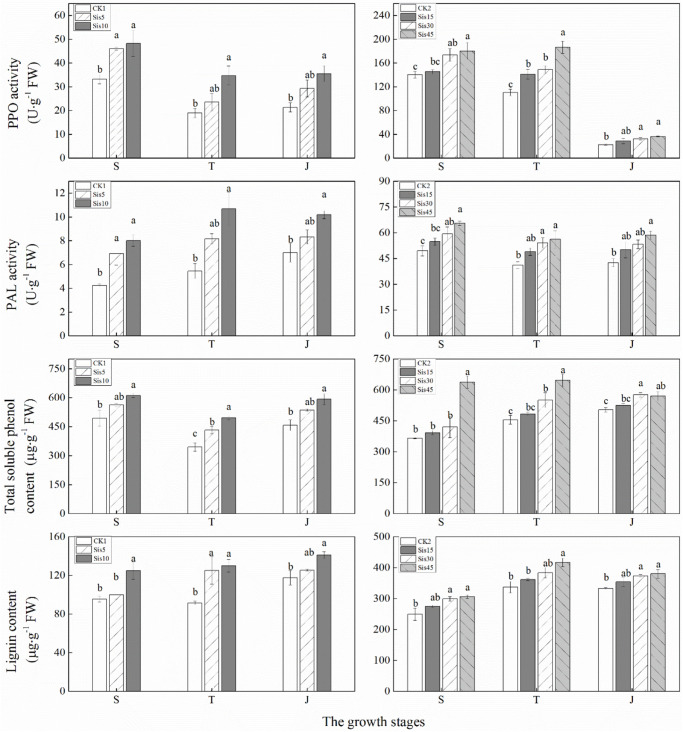
Effects of silicon application on secondary metabolism-related enzymes activities [polyphenoloxidase (PPO) and phenylalanine-ammonia-lyase (PAL)] and the contents of secondary metabolites (total soluble phenol and lignin) in sugarcane leaves under *Sporisorium scitamineum* stress. Various lowercase letters represent significant differences at the *P* level of 0.05. S, seedling stage; T, tillering stage; J, jointing stage.

In ROC22, the PPO activity of the three treatments initially decreased and then slightly increased with growth, whereas PAL activity changed relatively smoothly (Figure 4). PPO and PAL activities in each treatment in the three growth stages increased with greater Si application. Compared with CK1, PPO and PAL activities in Sis10 in the three growth stages and Sis5 at the seedling stage significantly increased (*P* ≤ 0.05).

In Badila, PPO activity continuously decreased, whereas PAL activity initially decreased and then stabilized (Figure 4). PPO and PAL activity in each treatment during the three stages increased with greater Si application. Compared with CK2, the PPO activity of Sis30 and Sis45 during the three stages and Sis15 at the tillering stage significantly increased (*P* ≤ 0.05). PAL activity in Sis45 during the three growth stages and Sis30 at the seedling and tillering stages significantly increased (*P* ≤ 0.05).

In the 2016 experiment, with growth, total soluble phenol content in the three treatments initially decreased and then increased, whereas lignin slightly increased (Figure 4). Total soluble phenol and lignin contents in each treatment during the three growth stages increased with greater Si application. Compared with CK1, total soluble phenol and lignin contents in Sis10 during the three growth stages and Sis5 at the tillering stage both significantly increased (*P* ≤ 0.05).

In the 2017 experiment, total soluble phenol content of the four treatments increased, and lignin content initially increased and then decreased with growth (Figure 4). Total soluble phenol and lignin contents in each treatment during the three growth stages increased with greater Si application. Compared with CK2, total soluble phenol content in Sis45 during the three growth stages and Sis30 at the tillering and jointing stages significantly increased (*P* ≤ 0.05). Lignin content in Sis45 during the three growth stages and Sis30 at the seedling and jointing stages significantly increased (*P* ≤ 0.05).

### Active Oxygen Metabolism-Related Enzymes and Metabolites in Sugarcane Leaves

**FIGURE 5:**
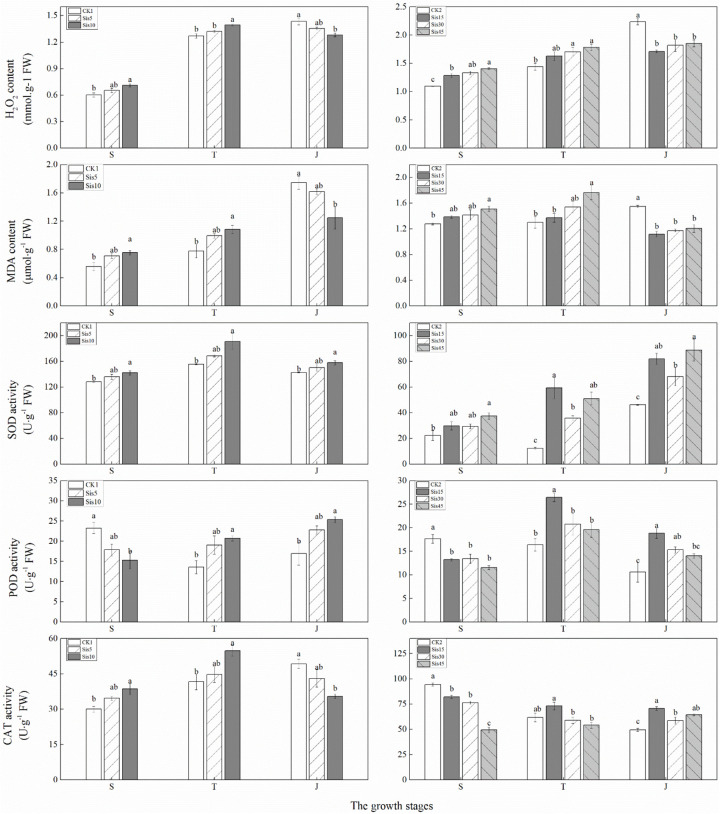
Effects of silicon application on malondialdehyde (MDA) and H_2_O_2_ levels and superoxide dismutase (SOD), peroxidase (POD), and CAT activity in sugarcane leaves under *Sporisorium scitamineum* stress. Various lowercase letters represent significant differences at a *P* level of 0.05. S, seedling stage; T, tillering stage; J, jointing stage.

In the 2016 experiment, with growth, H_2_O_2_ and MDA levels in the leaves without Si treatment (CK1) continuously increased, whereas that of H_2_O_2_ in the leaves with Si treatment (Sis5 and Sis10) initially increased and then decreased, whereas that of MDA continuously increased (Figure 5). H_2_O_2_ and MDA levels at the seedling and tillering stages increased with greater Si application, whereas that at the jointing stage decreased with greater Si application. Compared with CK1, H_2_O_2_ and MDA levels in Sis10 at the seedling and tillering stages significantly increased (*P* ≤ 0.05), whereas those in Sis10 at the jointing stage significantly decreased (*P* ≤ 0.05).

In the 2017 experiment, with growth, H_2_O_2_ content in the leaves without Si treatment (CK2) continuously increased, whereas MDA content was initially stable and then increased. H_2_O_2_ levels with Si treatment (Sis15, Sis30, and Sis45) initially increased and then stabilized, whereas MDA levels initially increased and then decreased (Figure 5). H_2_O_2_ and MDA levels at the seedling and tillering stages increased with greater Si application, whereas those at the jointing stage decreased with greater Si application. Compared with CK2, H_2_O_2_ levels at the seedling and tillering stages of Si treatment significantly (*P* ≤ 0.05) increased (except for Sis15 at the tillering stage), and MDA content in Sis45 at the seedling and tillering stages significantly increased (*P* ≤ 0.05), whereas H_2_O_2_ and MDA levels with Si treatment at the jointing stage significantly decreased (*P* ≤ 0.05).

In ROC22, with growth, SOD activity in the leaves of the three treatments (CK1, Sis5, and Sis10) initially increased and then decreased. CAT activity in the leaves without Si treatment continuously increased, whereas that of the leaves with Si treatment initially increased and then decreased. POD activity in the leaves without Si treatment initially decreased and then increased, whereas that of leaves with Si treatment steadily increased (Figure 5). SOD activity during the three growth stages increased with greater Si application. CAT activity at the seedling and tillering stages increased with greater Si application, whereas that at the jointing stage decreased with greater Si application. POD activity decreased with greater Si application at the seedling stage, and increased with greater Si application at the tillering and jointing stages. Compared with CK1, SOD activity in Sis10 during the three growth stages significantly increased (*P* ≤ 0.05). CAT activity of Sis10 at the seedling and tillering stages significantly increased (*P* ≤ 0.05), whereas that of Sis10 at the jointing stage significantly decreased (*P* ≤ 0.05). POD activity in Sis10 at the tillering and jointing stages significantly increased (*P* ≤ 0.05), whereas POD activity in Sis10 at the seedling stage significantly decreased (*P* ≤ 0.05).

In Badila, with growth, SOD activity in the leaves without Si treatment initially decreased and then increased, whereas that of leaves with Si treatment increased. CAT activity of the leaves without Si application (CK2) and low Si application (Sis15) continuously decreased, that of leaves with medium Si application (Sis30) initially decreased and then increased, and that of leaves with high Si application (Sis45) increased. POD activity in leaves without Si treatment decreased, whereas that in leaves with Si treatment initially increased and then decreased (Figure 5). SOD activity increased with greater Si application during the three growth stages, CAT and POD decreased with greater Si application at the seedling stage, and CAT and POD activity initially increased and then decreased with greater Si application at the tillering and jointing stages. Compared with CK2, the SOD activity of leaves with Si treatment (Sis15, Sis30, and Sis45) at the tillering and jointing stages significantly increased (*P* ≤ 0.05), whereas only Sis45 significantly increased (*P* ≤ 0.05) at the seedling stage. CAT activity with Si treatment at the seedling stage significantly decreased (*P* ≤ 0.05); SOD activity with Si treatment at the jointing stage significantly increased (*P* ≤ 0.05). POD with Si application at the seedling stage significantly decreased (*P* ≤ 0.05), whereas that in Sis15 at the tillering stage and Sis15 and Sis30 at the jointing stage significantly increased (*P* ≤ 0.05).

## Discussion

Si plays an active role in plant disease prevention and control, improving plant resistance to disease and in turn reducing disease incidence. Ge et al. (2014) studied the effect of Si application on the control of rice blast using a Hoagland nutrient solution hydroponic test, which showed that Si significantly reduced rice blast in two resistant and susceptible rice lines and increased resistance to rice blast. Liu et al. (2016) studied the effect of Si on resistance to rice blight by inoculating *Xanthomonas oryzae* pv. *oryzae*, and the results showed that the disease index with Si treatment was 17.8% lower than that of non-Si treatment. There are few reports on the control of sugarcane diseases by Si. Si application is beneficial to control sugarcane ringspot (*Leptosphaeria sacchari* Breda de Hann) (Raid et al., 1992) and can reduce the severity of sugarcane brown rust (*Puccinia melanocephala*) (Naidoo et al., 2009). In this study, smut incidence was decreased by 11.57–22.58% (ROC22) and 27.75–46.67% (Badila) through 2 years of Si application. At the same time, the incidence of smut was negatively correlated with the amount of Si applied and Si content in the leaves, stems, and roots of sugarcane plants, indicating that Si has a significant effect on sugarcane smut, and the control effect increased with greater Si content. Winslow et al. (1997) obtained similar results in relation to rice diseases, i.e., the relationship between the severity of disease and Si content in plant tissues is negatively correlated. In this study, the correlation between Si content in leaves or stems of different sugarcane varieties (ROC22 or Badila) and the incidence of sugarcane smut was different. The main reason is that there are only two sugarcane varieties participating in this test, so it is necessary to expand the number of varieties in future experiments. In addition, our study also found that treatment with a high amount of Si (Sis45) inhibited the growth of sugarcane (Supplementary Figure S1), and thus the application of appropriate amount of Si could promote sugarcane growth. Therefore, the application of the appropriate amount of Si effectively prevents and controls sugarcane smut, as well as promotes sugarcane growth.

When plants are infected by pathogens, these are often induced to produce pathogenesis-related proteins (PR proteins, a class of small-molecule proteins) to resist further infection (Brogue et al., 1991). Chitinase and β-1,3-glucanase are two important types of PR proteins that are widely distributed in higher plants. In general, the expression levels of the two PR proteins are relatively low, and their content and activity can be rapidly increased to enhance resistance to adverse external stimuli. Su et al. (2013, 2014) found that both chitinase and β-1,3-glucanase were induced and positively correlated with sugarcane smut resistance after *S. scitamineum* infection. In this study, the activity of chitinase and β-1,3-glucanase in the leaves under *S. scitamineum* stress significantly increased with greater Si application and can maintain high activity at the three growth stages, which indicated that Si can significantly increase the activity of chitinase and β-1,3-glucanase in leaves to enhance smut resistance upon infection with *S. scitamineum*. Xue et al. (2010) also reported similar findings that Si application rapidly increased chitinase and β-1,3-glucanase activity in rice and enhanced resistance to *X. oryzae* pv. *oryzae*.

Secondary metabolism is the process of biosynthesizing non-essential substances in life and storing secondary metabolites. PPO and PAL are secondary metabolism-related enzymes, whereas phenols and lignin are the main secondary metabolites. The higher the PPO and PAL activity in plants, the stronger the disease resistance (Schneider and Ullrich, 1994; Wang et al., 2001). Phenolic substances and lignin can form a physical antimicrobial barrier and enhance plant disease resistance (Vance et al., 1980; Bonello and Pearce, 1993). In this study, Si application significantly increased PPO and PAL activity and total soluble phenol and lignin levels in sugarcane leaves to reduce smut incidence and enhance smut resistance under *S. scitamineum* stress. Sun et al. (2009) found that Si significantly increases PPO and PAL activity in rice under rice blast stress, as well as increase disease resistance, which is similar to the results of this study. Fawe et al. (1998) first reported that Si may be involved in the secondary metabolism of cucumber and promotes the increase of secondary metabolites such as flavonoids to increase resistance to powdery mildew (*Sphaerotheca fuliginea*). This study hypothesized that Si participates in the regulation of secondary metabolism of sugarcane under *S. scitamineum* stress, which in turn positively regulates the activities of secondary metabolic enzymes such as PPO and PAL and the synthesis of secondary metabolites such as total soluble phenol and lignin to increase sugarcane resistance to smut. Additionally, the elevated lignin content may provide an evidence for the hypothesis that Si strengthens plant cells as a physical barrier.

Under normal circumstances, the elimination of antioxidant enzyme systems (e.g., CAT, SOD, and POD) in plant cells and the production of reactive oxygen species (ROS) (such as MDA and H_2_O_2_) are in dynamic equilibrium. ROS metabolism is disrupted by stress, causing its accumulation or exacerbating lipid peroxidation, which in turn leads to cell plasma membrane damage. MDA is the main product of membrane lipid peroxidation, and its content reflects the degree of membrane lipid peroxidation (Mittler, 2002). H_2_O_2_ can directly or indirectly disrupt membrane lipid peroxidation and can also rapidly accumulate in cells to induce plant resistance (Torres et al., 2006). It has been reported that Si can regulate ROS levels to improve the ability to resist disease (Künstler et al., 2015). Si can significantly increase ROS levels in rice leaves to increase resistance to bacterial wilt (*X. oryzae* pv. *oryzae*) and sheath blight (*Rhizoctonia solani*) (Rodrigues et al., 2003; Xue et al., 2010). In this study, MDA and H_2_O_2_ levels in the leaves at the seedling and tillering stages with Si treatment were significantly higher than those without Si under *S. scitamineum* stress, whereas those in the jointing stage significantly decreased. We hypothesize that Si regulates the accumulation of ROS to stimulate the resistance response during early growth stages (seedling and tillering stages). In addition, ROS levels in the later stage significantly decreased with the ROS metabolism enzymes acting. Xue et al. (2010) determined that Si significantly increases ROS content in rice under bacterial blight stress, causing hypersensitive responses to increase resistance, which is concordant with the results of our study.

Superoxide dismutase can scavenge superoxide radicals and cooperate with other antioxidant enzymes (e.g., POD and CAT) to prevent the cell membrane damage due to ROS or peroxides. CAT and POD are defensive enzymes that can clear H_2_O_2_. In this study, the SOD activity of Si-treated leaf was higher than that of non-Si-treated (CK1 and CK2) at the three stages (the treatment with high amounts of Si reached significant levels) under *S. scitamineum* stress, suggesting that the application of Si improves SOD activity in sugarcane leaves, promotes the removal of ROS, and reduces damage to plants due to *S. scitamineum* stress. In addition, in this study, the POD activity of sugarcane leaves treated with Si was significantly lower in the early stage (seedling stage) than that of sugarcane leaves treated without Si, which allowed the accumulation of H_2_O_2_ to stimulate the defense response of sugarcane plants. The increase in POD activity during the later growth stage in sugarcane can remove excessive ROS to protect the plants and improve resistance to smut.

Several reports have shown that Si increases the resistance of plants to diseases; however, the underlying mechanism of disease resistance remains unclear. One suggestion is that Si acts as a physical barrier, i.e., Si is deposited in the plant cell wall, resulting in silicified cells, thereby effectively preventing the invasion of fungal mycelia, and at the same time alleviating the effect of enzymes secreted by fungi on the cell wall (Yoshida, 1965; Chinnoi and Christopher, 2011). Some think that it is biochemical defense, i.e., Si induces a signal transduction substance that participates in the physiological and biochemical process of interaction between plants and pathogens, thereby improving the resistance of plants to diseases (Cherif et al., 1994). Others believe that the two mechanisms work together (Liang et al., 2007). This study is mainly based on the physiological and biochemical aspects of the mechanism of Si in improving the resistance of sugarcane to smut, while the physical barrier is limited (only the correlation between Si content in roots, stems, and leaves of sugarcane plants and the incidence of smut were analyzed), which needs further study. In addition, field plot test is also needed to further verify the control effect of Si application on sugarcane smut.

## Conclusion

This study showed that Si application increases smut resistance in sugarcane, with smut incidence decreasing to 11.57–22.58% in ROC22 and 27.75–46.67% in Badila. Smut incidence is negatively correlated with the amount of Si applied and the Si content in the leaves, stems, and roots of sugarcane plants (highly significantly negatively correlated with the Si content in stems). Si is involved in the physiological and biochemical processes of sugarcane, such as secondary metabolism, ROS metabolism, and pathogenesis-related protein activity regulation, and positively regulates smut resistance in sugarcane. This study is the first to reveal the physiological mechanism of Si in improving smut resistance in sugarcane, as well as provides a theoretical basis for the development of Si fertilizers to control sugarcane smut.

## Data Availability Statement

All datasets presented in this study are included in the article/Supplementary Material.

## Author Contributions

WS conceived and designed the experimental plan. QD and JW performed the experiments. QD analyzed the data and wrote the manuscript. WS and JC revised the manuscript. All authors read and approved the final version of the manuscript.

## Conflict of Interest

The authors declare that the research was conducted in the absence of any commercial or financial relationships that could be construed as a potential conflict of interest.
