# Supplementary material for: Physiological Mechanisms of Improved Smut Resistance in Sugarcane Through Application of Silicon
Source: Front Plant Sci. 2020 Nov 5;11:568130. doi: 10.3389/fpls.2020.568130 (PMC7674639; doi:10.3389/fpls.2020.568130)
Supplement: Supplementary file 1 [file Image_1.pdf]

Supplementary material

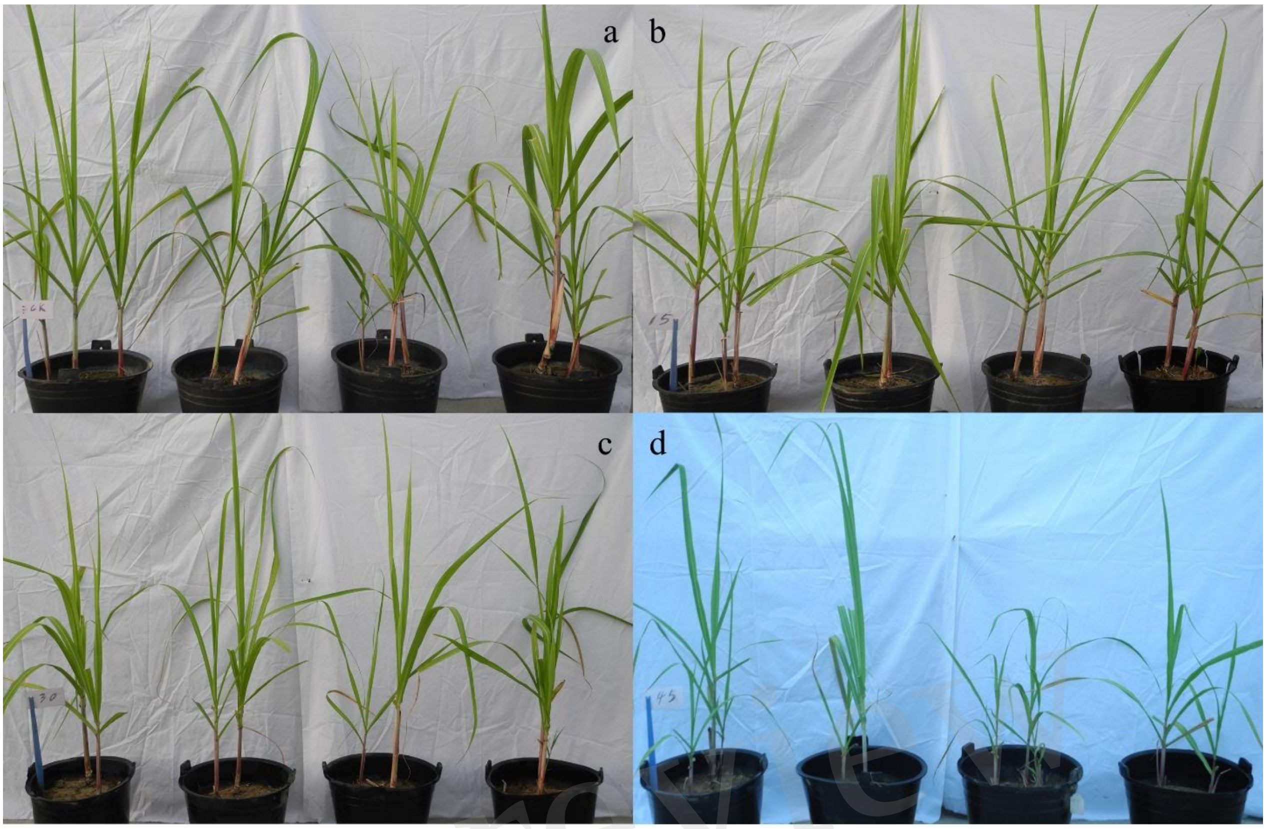

Figure S1 Effect of silicon application on the growth of Badila.

a, CK2, no ESi application; b, Si15, 15g ESi barrel $^{-1}$; c, Si30, 30g ESi barrel $^{-1}$; d, Si45, 45g ESi barrel $^{-1}$

In review
